# Suicidal ideation in Chinese patients with advanced breast cancer: a multi-center mediation model study

**DOI:** 10.1186/s40359-024-01607-x

**Published:** 2024-03-12

**Authors:** Yening Zhang, Yi He, Ying Pang, Zhongge Su, Yu Wang, Yuhe Zhou, Yongkui Lu, Yu Jiang, Xinkun Han, Lihua Song, Liping Wang, Zimeng Li, Xiaojun Lv, Yan Wang, Juntao Yao, Xiaohong Liu, Xiaoyi Zhou, Shuangzhi He, Lili Song, Jinjiang Li, Bingmei Wang, Lili Tang

**Affiliations:** 1 Key Laboratory of Carcinogenesis and Translational Research (Ministry of Education/Beijing), Department of Psycho-oncology, Peking University Cancer Hospital &Institute, Fu-Cheng Road 52, Hai-Dian District, 100142 Beijing, China; 2 Department of Breast Cancer Radiotherapy, Cancer Hospital, Chinese Academy of Medical Sciences, Shanxi Medical University, Taiyuan, China; 3 The Fifth Department of Chemotherapy, The Affiliated Cancer Hospital of Guangxi Medical University, Guangxi Zhuang Autonomous Region, Nanning, China; 4 Department of Medical Oncology, Cancer Center, West China Hospital, Sichuan University, Chengdu, China; 5 Department of Breast Medical Oncology, Shandong Cancer Hospital and Institute, Shandong First Medical University, Shandong Academy of Medical Sciences, Jinan, China; 6 Department of Oncology, the First Affiliated Hospital of Zhengzhou University, Zhengzhou, China; 7 Department of Oncology, Xiamen Humanity Hospital, Xiamen, China; 8 Department of Integrated Chinese and Western Medicine, Shaanxi Provincial Cancer Hospital Affiliated to Medical College of Xi’an Jiaotong University, Xi’an, China; 9 Department of Clinical Spiritual Care, The Affiliated Cancer Hospital of Xiangya School of Medicine, Hunan Cancer Hospital, Central South University, Changsha, China; 10 Radiotherapy Center, Hubei Cancer Hospital, Wuhan, China; 11 Department of Psycho-oncology, Shandong Cancer Hospital and Institute, Shandong First Medical University, Shandong Academy of Medical Sciences, Jinan, China

**Keywords:** Suicidal ideation, Advanced breast cancer, Mediation analysis, Psychological distress, Symptom burden

## Abstract

**Purpose:**

The pathways underpinning suicide ideation (SI) and certain physical and psychological factors in patients with advanced breast cancer remain unclear. This study develops and validates a mediation model that delineates the associations between several multidimensional variables and SI in Chinese patients with advanced breast cancer.

**Methods:**

Patients with advanced breast cancer (*n* = 509) were recruited as study participants from 10 regional cancer centers across China from August 2019 to December 2020. Participants were required to complete five questionnaires using an electronic patient-reported outcomes (ePRO) system: 9 item- Patient Health Questionnaire (PHQ-9), Hospital Anxiety and Depression Scale (HADS), Insomnia Severity Index (ISI), 5-level EQ-5D (EQ-5D-5L), and MD Anderson Symptom Inventory (MDASI). Risk factors for SI were identified using multivariable logistic regression, and inputted into serial multiple mediation models to elucidate the pathways linking the risk factors to SI.

**Results:**

SI prevalence was 22.8% (116/509). After adjusting for covariates, depression (odds ratio [OR] = 1.384), emotional distress (OR = 1.107), upset (OR = 0.842), and forgetfulness (OR = 1.236) were identified as significant independent risk factors (all *p* < 0.05). The ORs indicate that depression and distress have the strongest associations with SI. Health status has a significant indirect effect (OR=-0.044, *p* = 0.005) and a strong total effect (OR=-0.485, *p* < 0.001) on SI, mediated by insomnia severity and emotional distress.

**Conclusions:**

There is a high SI prevalence among Chinese patients with advanced breast cancer. Our analysis revealed predictive pathways from poor health to heightened SI, mediated by emotional distress and insomnia. Regular management of distress and insomnia can decrease suicide risk in this vulnerable population.

## Introduction

Suicide is one of the major causes of premature death worldwide. Data from the World Health Organization (WHO) indicates that more than 700,000 people died by suicide in 2019 globally, and 77% of these suicides occurred in low- and middle-income countries [1]. Reducing suicide rates via effective evidence-based interventions is a critical goal of the United Nations (UN) and the WHO. Suicidal ideation is a broad concept that describes thoughts of pursuing death, including active suicide ideation (experiencing current and specific suicide thoughts) and passive suicide ideation (a general wish to die but with no plan). One of the challenges to reducing suicide rates via clinical approaches is the lack of a consistent definition and gold standard for assessing and managing such strategies [2].

As indicated by a systematic review and meta-analysis study, cancer– as a life-threatening disease– can increase suicide risk in both sexes, and the increased pooled standardised mortality ratio (SMR) varies with the cancer site; SMR was 1.71 in breast cancer [3]. The findings of a population-based study indicate that suicide risk increases with an observed or expected ratio of 2.52 in patients who have received a cancer diagnosis within a year [4]. Kim et al. propose that 10.9% and 11.4% of breast cancer survivors presented with suicidal ideation at one week and at one year, respectively, after breast surgery; suicide ideation was linked to anxiety, depression and physical disability, brain-derived neurotrophic factor (BDNF) and the stage of the cancer [5]. Living in rural areas, experiencing severely traumatic events, being in paid employment, and having post-traumatic stress disorder (PTSD) have been verified to be associated with suicidal ideation in early-stage breast cancer [6]. A large sample analysis in the Surveillance, Epidemiology, and End Results Program (SEER) database reports that female breast cancer survivors have a higher risk of suicide than the general population (SMR = 1.19), and age group, race, marital status, cancer stage, radiotherapy, and molecular subtypes are independent predictors of suicide in this population [7]. Major depression disorder (MDD) is considered the main independent risk factor and a major effective treatment component for suicide [8, 9]. Research in India indicates that suicidal ideation in breast cancer patients is associated with impaired social emotion recognition and deficient social support [10]. Having suicidal ideation does not mean that a suicide attempt is certain, nor does it certainly result in suicide death. Nonetheless, a strong association between suicidal ideation at its worst point and suicide attempts has been verified [11], and there is no significant difference between the strengths of the correlation between suicide and suicidal ideation and the correlation between suicide and suicide behaviours [12]. However, identifying suicidal ideation and initiating rational preventive interventions present opportunities for saving lives [13].

Breast cancer is the most serious disease type affecting women’s health in China, ranking first in prevalence and fourth in mortality rate [14, 15]. The national cancer mortality trends in China changed between 1987 and 2020, with female breast cancer mortality increasing. This current trend demands that a high priority be placed on female breast cancer in public health policy formulation and cancer care [16]. Depression is the primary mood symptom presented by diagnosed cancer patients and is significantly associated with cancer recurrence, all-cause mortality, and cancer-specific mortality in breast cancer patients [17]. The severe symptoms subtype has the highest ratios in the 45–59 age group among breast cancer patients [18]. However, only a few studies have focused specifically on the incidence of suicide among breast cancer patients in China, as the general consensus has been that addressing depression fully mitigates suicidal tendencies in cancer patients. In a multicentre cross-sectional study, a noteworthy degree of suicidal ideation was recognised in advanced cancer patients without MDD [19]. This finding indicates that we should not limit the attention placed on suicidal ideation to individuals with major depressive disorder. We reanalyse certain characteristics of the breast cancer subgroup in our study sample to: (1) explore the prevalence of suicidal ideation in patients with advanced breast cancer, (2) address independent risk factors in this population, and (3) develop a predicting or mediating model that delineates the relationship between symptom burden or health status and suicidal ideation among this population.

## Methods

### Study participants and research setting

We conducted a national, multicentre cross-sectional study on advanced cancer patients (patients with the top six cancers in terms of high prevalence in China, including breast cancer) from August 2019 to December 2020. A subgroup of these advanced breast cancer patients was used for the analyses in this study. The patients were recruited as study participants from inpatient departments at 10 regional cancer centres in China, including centres in North China (Beijing and Shanxi), East China (Shandong and Fujian), South China (Guangxi), Central China (Hubei, Hunan and Henan), Northwest China (Shaanxi), and Southwest China (Sichuan). The inclusion criteria were as follows: diagnosed with advanced breast cancer, age ≥ 18 years old, and able to give informed consent. Patients with major cognitive impairment, communication difficulties, or a poor physical status that interfered with filling out the study questionnaires were excluded from the study. The study was approved by the Institutional Review Board of the Peking University Cancer Hospital on 14 May 2019 (approval number: #2019YJZ34).

### Study procedure

**Fig. 1:**
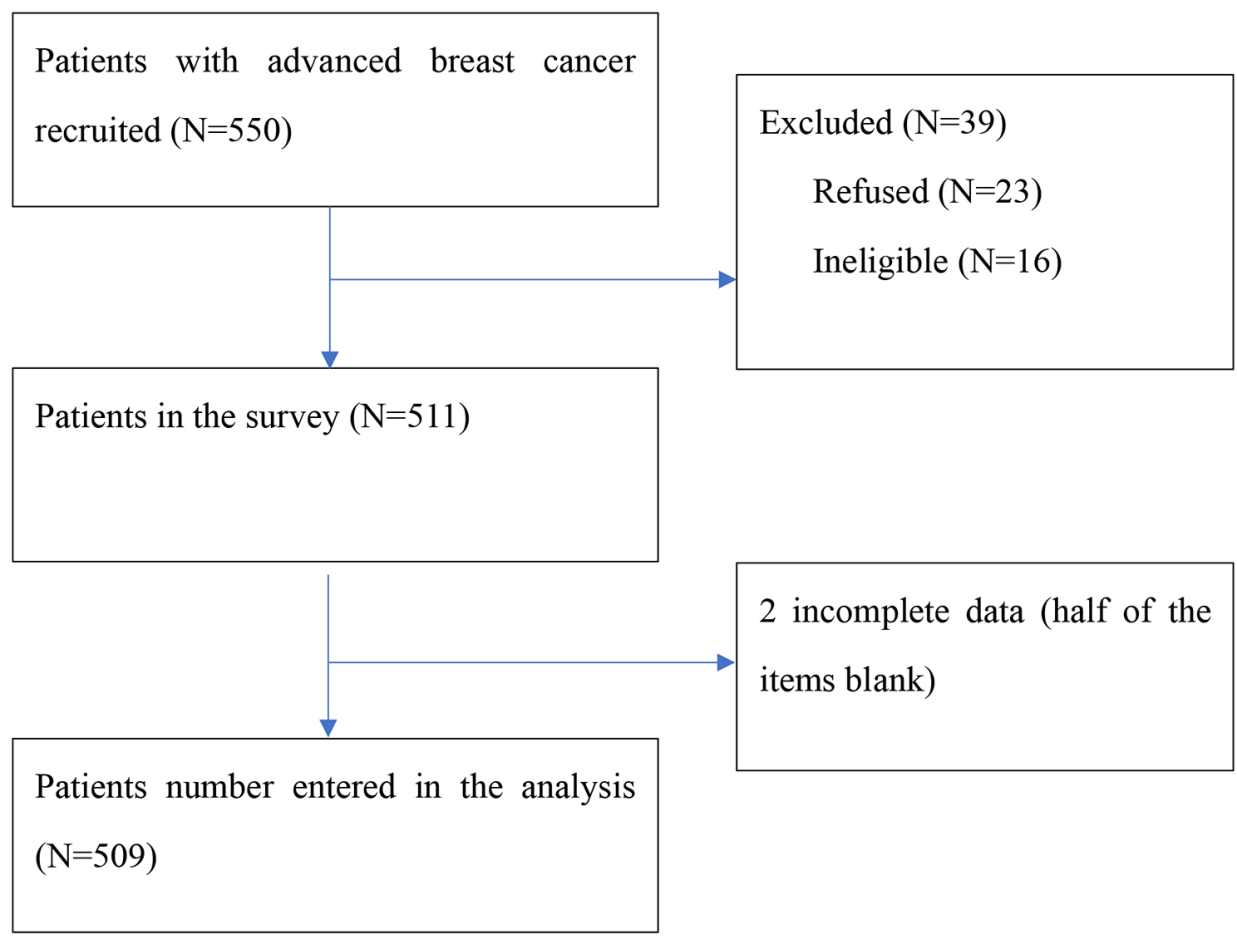
Flowchart in this study

Electronic patient-reported outcomes (ePRO) were captured using validated assessment tools: social demographic and medical information, depression severity and suicide ideation were assessed using Item 9 of the Patient Health Questionnaire (PHQ-9); emotional distress was assessed using the Hospital Anxiety and Depression Scale (HADS); insomnia severity was measured using the Insomnia Severity Index (ISI); health status was evaluated using the five-level EQ-5D (EQ-5D-5L); and symptom burdens and interference were assessed using the M. D. Anderson Symptom Inventory (MDASI). The data were captured using iPads on which the ePRO system was loaded, and this study is the first time an ePRO has been applied in a national, multicentre study. Oncologists and investigators at the 10 recruiting sites received unified on-site and online training and competency assessments before the data collection exercise. All data were collected during patient hospitalisation and uploaded to the central database in real time. Severe physical and psychological symptoms and positive suicide ideation (indicated by an Item 9 score of ≥ 1 on the PHQ-9) prompted further assessment or immediate intervention by the local multidisciplinary supportive care team comprising subsets of oncologists, oncology nurses, psychiatrists, psychologists, and/or social workers– depending on local availability at each site. Participant data with more than half of the item scores missing were deleted from the database before statistical analysis was performed (Fig. 1). To mitigate potential sources of bias, we implemented several strategies in this cross-sectional study: standardised training procedures (more than three training sessions were conducted, both on-site and online); consecutive enrolment of all eligible advanced breast cancer patients during the study period to minimise selection bias at each site; use of validated measurements to enhance reliability and accuracy in the assessment of all variables; collection of key sociodemographic and clinical data from the medical records to control for potential confounding factors.

### Measurements

#### The MD Anderson symptom inventory (MDASI)

The MDASI is widely used for patient-reported outcomes on symptom burden and interference experienced by cancer patients over the last 24 h. The core MDASI comprises 13 symptom items and six interference items that are generally applicable to all kinds of cancer patients. Each item is scored on a scale of 0 (not present) to 10 (as bad as you can imagine). The Chinese version of the core MDASI has been proven to have good reliability and validity [20]. For breast cancer patients, in addition to the core MDASI items, we added three items (i.e., constipation, hot flush and oedema) based on the same scoring algorithm. Based on the MDASI user guide, an item a score of ≥ 5 on a scale item indicates a moderate symptom burden, while a score of ≥ 7 indicates a severe symptom burden.

#### Hospital anxiety and depression scale (HADS)

The HADS is used to assess the emotional distress a patient has experienced over the past two weeks. The scale has 14 items (seven items for anxiety and seven items for depression). Each item is scored on a scale of 1 to 4 points, with a total score ranging from 14 to 56. Items 2, 4, 7,10 and 14 use reverse scoring. The Chinese version of the HADS has good reliability and validity when applied to general outpatients [21]. Clinically significant emotional distress is indicated by a total HADS score of ≥ 15, and anxiety and depression are indicated by a score of ≥ 10 on the HADS anxiety subscale and depression subscales, respectively.

#### Patients health questionnaire (PHQ-9) item 9

The PHQ-9 is a nine-item self-report scale used to assess depression over the past two weeks in primary care settings [22] and among cancer patients [23]. Each item is scored on a scale of 0 (absolutely no) to 3 (nearly every day), with a total score ranging from 0 to 27. A total score of ≥ 10 indicates moderate and severe depression. The psychometric value of the Chinese version of the PHQ-9 has been verified for the primary care population [24]. Suicidal ideation (SI) was determined based on the scores on Item 9 of the PHQ-9, which asks the question, ‘How often have you been bothered by thoughts about being better off dead or of hurting yourself in some way over the past two weeks?’. A score of ≥ 1 was classified as indicating that the participant was experiencing SI, as previous studies have demonstrated the acceptable accuracy of this single item against specific suicide assessment tools, such as the Columbia Suicide Severity Rating Scale [25], and such a score on Item 9 of the PHQ-9 is associated with suicide attempts [26].

#### Insomnia severity index (ISI)

The ISI has seven items for assessing the severity and impact of insomnia experienced by patients over the past two weeks. Each item is scored on a scale of 0 (no problem) to 4 (very severe), with the total score ranging from 0 to 28. A score of 15 has been validated as the cutoff value for moderate to severe insomnia. The Chinese version of the ISI has been validated for older community-dwelling adults and patients with insomnia [27, 28].

#### Five-level EuroQol five-dimensional questionnaire (EQ-5D-5L)

The EQ-5D-5L is developed by the EuroQol Group and widely used for health status assessment. The questionnaire includes a five-dimensional descriptive system (mobility, self-care, usual activities, pain/discomfort, anxiety/depression) and the EuroQol visual analogue scale (EQ-VAS). Each dimension is scored on a five-level scale (1 to 5), and the EQ-VAS is scored on a scale of 0 to 100. A Chinese value set has been generated using a sample of urban residents [29].

The Eastern Cooperative Oncology Group performance status (ECOG PS) scale is a six-point scale ranging from 0 (normal with full function) to 5 (dead), with low scores indicating a good performance status. The ECOG PS is commonly used to determine whether a patient can receive chemotherapy and to predict survival outcomes [30].

### Statistical analysis

To evaluate normality, histograms and quantile–quantile (Q-Q) plots were visually examined, and quantitative normality tests were conducted on all continuous variables. The Kolmogorov–Smirnov and Shapiro–Wilk tests were used to quantitatively assess normality. Non-normally distributed variables were analysed using nonparametric methods. The two-sided t-test was used for between-group comparison of two mean values when the continuous variables had a normal distribution, and the Cohen’s d value was reported as the effect size indicator (small: ≥0.2, medium: ≥0.5, large: ≥0.8) [31]. When the continuous variables were a non-normal distribution, the nonparametric Chi-square test and the Kruskal–Wallis H test were used for between-group comparisons, and the effect size indicators used were Cramér’s V (small: ≥0.1, medium: ≥0.3, large: ≥0.5) and Epsilon-squared (ε^2^; small: ≥0.01, medium: ≥0.06, large: ≥0.14) [32, 33]. Univariate and multivariable logistic regression analyses were used to determine the independent risk factors for SI in the study sample. These statistical analyses were performed using the IBM Statistical Package for the Social Sciences (SPSS) 29.0.

A mediation analysis was performed to test for indirect effects, with R^2^ reported as the effect size indicator (small: ≥0.02, medium: ≥0.13, large: ≥0.26) [34]. Serial multiple mediation analyses were then performed using R 4.2.2. The bootstrapping method was used to produce 95% bias-corrected confidence intervals (CIs) for the magnitude of the indirect effects based on 1,000 resamples of the data. The CIs that do not include zero indicate effects that are significant, and a total estimate effect of ≥ 0.3 or ≤ − 0.3 means acceptable [35].

## Results

### Descriptive results of demographic and medical data

Five hundred and nine breast patients completed the ePRO assessment, with a response rate of 92.5% (509/550). The average age of the individuals in the study sample was 50.34 ± 9.996. Most of the study participants were married, 326 participants (67.2%) had remote metastatic breast cancer, and 386 (76.3%) had experienced less than 5% weight loss over the preceding six months. Nearly two-thirds of the participants had received chemotherapy (346/433, 79.9%) and surgery (294/435, 67.6%) and had been undergoing anticancer treatment (389/489, 79.6%). Notably, 22.8% (116/509) of this study sample had experienced SI. The results of a between-group comparison of the demographic and medical information on patients with and without SI are presented in Table 1. We found no disparity between these two groups except in their ECOG PS scores (χ^2^ = 6.411, *p* = 0.041, Cramér’s V = 0.113) and cancer stage (χ^2^ = 7.811, *p* = 0.005, Cramér’s V = 0.124). This information is also presented in Table 1.

**Table 1:** demographic and medical information in patients with and without suicide ideation (SI)

Variables	Total	Group without SI (*n* = 389) M ± SD/n (%)	Group with SI (*n* = 116) M ± SD/n (%)	t /χ^2^	p	Effect size Cohen’s d/ Cramer’s V
Age	50.34 ± 9.996	50.12 ± 9.762	51.27 ± 10.481	-1.090	0.276	-0.115 (Cohen’s d)
18–45	168(33.5)	128(33.2)	39(33.6)	3.650	0.161	0.085
46–64	294(58.6)	231(60.0)	63(54.3)			
≥65	40(8.0)	26(6.8)	14(12.1)			
Education				4.555	0.103	0.097
Middle school and under	254(51.9)	191 (50.7)	62(55.9)			
High and professional school	139(28.4)	104 (27.6)	35(31.5)			
College and above	96(19.6)	82(21.8)	14(12.6)			
ECOG				6.411	0.041*	**0.113**
0	189(37.4)	150 (38.6)	39(33.6)			
1	240(47.4)	189(48.6)	51(44.0)			
2	77(15.2)	50(12.9)	26(22.4)			
Weight loss				0.339	0.561	0.026
Weight loss<5%	386(76.3)	295(75.8)	91(78.4)			
Weight loss ≥ 5%	120(23.7)	94(24.2)	25(21.6)			
Marital status				0.079	0.779	0.045
Without partner	32(6.3)	27(6.9)	5(4.3)			
Married	474(93.7)	362(93.1)	111(95.7)			
Job situation				1.042	0.307	0.048
Full time	244 (48.2)	193(49.6)	51 (44.0)			
Part time and unemployed	262(51.8)	196(50.4)	65 (56.0)			
Cancer stage				7.811	0.005**	**0.124**
Cancer in situ/local metastasis	159(32.8)	126(32.4)	54(46.6)			
Remote metastasis	326(67.2)	263(67.6)	62(53.4)			
Income (RMB/month)				0.441	0.932	0.032
<3000	134(30.2)	101(29.4)	32(32.0)			
≥3000, <6000	137(30.9)	107(31.2)	30(30.0)			
≥6000, <10,000	83(18.7)	66(19.2)	17(17.0)			
≥10,000	90(20.3)	69(20.1)	21(21.0)			
Quantity of living persons				0.112	0.739	0.015
3 and under	290(59.8)	220(59.3)	69(61.1)			
4 and above	195(40.2)	151(40.7)	44(38.9)			
Anti-cancer treatment now				0.614	0.433	0.035
No	91 (18.6)	87(22.4)	30(25.9)			
Yes	389(79.6)	302(77.6)	86(74.1)			
Using opioid				0.891	0.345	0.042
No	476(94.1)	368(94.6)	107(92.2)			
Yes	30(5.9)	21(5.4)	9(7.8)			
Using anti-vomiting drugs				0.655	0.418	0.036
No	445 (87.4)	343(88.2)	99(85.3)			
Yes	64 (12.6)	46(11.8)	17(14.7)			
Using endocrine drugs				1.000	0.317	0.045
No	465(91.9)	360(92.5)	104(89.7)			
Yes	41(8.1)	29(7.5)	12(10.3)			

***p<0.001; **p<0.01, *p<*0.05*

Cohen’s d: small ≥ 0.2, medium ≥ 0.5, large ≥ 0.8

Cramer’s V: small ≥ 0.1, medium ≥ 0.3, large ≥ 0.5

### Comparison of physical and psychological variables between patients with and without SI

In our study sample, we found disparities between participants with SI and those without SI regarding all the psychological and symptom variables considered. Participants with SI had significantly higher scores than participants without SI on psychological distress (χ^2^ = 100.286, *p* < 0.001), major depression (χ^2^ = 116.924, *p* < 0.001), and insomnia (χ^2^ = 13.623, *p* < 0.001), as well as a lower health status (EQ-5D-5L value, t = 4.987, *p* < 0.001; EQ-5D-5L VAS: t = 5.258, *p* < 0.001). The effect size for these differences was small to medium, with the Cramér’s V values ranging from 0.164 (indicating insomnia on the ISI) to 0.481 (indicating major depression on the PHQ-9). The top five symptoms with a moderate symptom burden among the study population were insomnia (22.1%), fatigue (21.6%), upset (17.4%), forgetfulness (17.2%), and loss of appetite (15.6%). In addition, when compared to participants without SI, a significantly higher proportion of the study participants with SI had fatigue scores of ≥ 5 (38.8% vs. 21.9%, χ^2^ = 13.303, *p* < 0.001, Cramér’s V = 0.162), upset scores of ≥ 5 (34.5% vs. 17.1%, χ^2^ = 113.975, *p* < 0.001, Cramér’s V = 0.167), and forgetfulness scores of ≥ 5 (29.9% vs. 18.1%, χ^2^ = 7.619, *p* = 0.006, Cramér’s V = 0.119), all indicating a medium effect size (Table 2).

**Table 2:** Discrepancy of physical and psychological variables between patients with and without suicide ideation (SI)

Variables	Total	Without SI (*n* = 372) M ± SD/n (%)	With SI(*n* = 79)M ± SD/n (%)	χ^2^	p	Effect size: Cramer’s V/ε^2^
HADS	11.47 ± 7.224	9.67 ± 6.366	16.92 ± 6.327	——	——	——
	HADS<15	307(78.9)	34(29.3)	100.286	<0.001***	0.446
	HADS ≥ 15	82(21.1)	82(70.7)			
PHQ-9	6.40 ± 4.821	5.04 ± 4.007	10.28 ± 3.860	——	——	——
	PHQ-9<10	342 (87.9)	46(39.7)	116.924	<0.001***	0.481
	PHQ-9 ≥ 10	47(12.1)	70(60.3)			
ISI	6.69 ± 6.101	5.91 ± 5.692	9.23 ± 6.386	——	——	——
	ISI<15	352 (90.5)	90(77.6)	13.623	<0.001***	0.164
	ISI ≥ 15	37 (9.5)	26 (22.4)			
Equation 5D5L value	0.819 ± 0.230	0.852 ± 0.196	0.708 ± 0.292	46.363	<0.001***	0.091(ε^2^)
Equation 5D5L VAS	71.66 ± 20.231	75.55 ± 19.031	62.98 ± 21.107	33.742	<0.001***	0.066(ε^2^)
MDASI-Fatigue	2.73 ± 2.594	2.37 ± 2.431	3.57 ± 2.709	——	——	——
	Fatigue<5	303 (78.1)	71 (61.2)	13.303	<0.001***	0.162
	Fatigue ≥ 5	85 (21.9)	45 (38.8)			
MDASI-Upset	2.36 ± 2.488	2.02 ± 2.335	3.28 ± 2.615	——	——	——
	Upset<5	319 (82.9)	76 (65.5)	13.975	<0.001***	0.167
	Upset ≥ 5	66 (17.1)	40 (34.5)			
MDASI-Forgetfulness	2.29 ± 2.394	2.01 ± 2.294	3.30 ± 2.545	——	——	——
	Forgetfulness<5	317 (81.9)	82 (70.1)	7.619	0.006**	0.119
	Forgetfulness ≥ 5	70 (18.1)	35(29.9)			

***p<0.001; **p<0.01, *p<*0.05*

Cramer’s V: small ≥ 0.1, medium ≥ 0.3, large ≥ 0.5

Epsilon-squared (ε^2^): small ≥ 0.01, medium ≥ 0.06, large ≥ 0.14

***Abbreviations***: *HADS-Hospital Anxiety and Depression Scale; PHQ-9-9 Item-Patients Health Questionnaire; ISI-Insomnia Severity Index; EQ-5D-5L- Five-level EuroQol five-dimensional questionnaire; MDASI- The MD Anderson Symptom Inventory*.

### Risk factors for suicidal ideation among Chinese patients with advanced breast cancer

Univariate and multivariable logistic regression analyses were used to explore the risk factors for SI among patients with advanced breast cancer. Factors with a significant correlation with SI were included in both the univariate and multivariable logistic regression analyses. The following factors were explored: cancer stage, performance status (assessed using the ECOG PS), emotional distress (assessed with the HADS), depression (assessed using the PHQ-9), insomnia (assessed using the ISI), health status (assessed using the EQ-5D-5 L and VAS), and symptom burden (assessed using the pain, fatigue, upset, and forgetfulness subscales of the MDASI). The results of the univariate logistic regression show that all the variables considered are risk factors for SI in the study population. Furthermore, cancer stage (odd ratio [OR] = 0.508, *p* = 0.016), emotional distress (OR = 1.090, *p* = 0.003), depression (OR = 1.355, *p*<0.001), and MDASI-forgetfulness (OR = 1.159, *p* = 0.016) were verified to be independent risk factors for SI in the study population (Table 3).

**Table 3:** Risk factors for suicide ideation in patients with advanced breast cancer patients: Results from univariate and multivariable Logistics regression

Variables	Univariate analysis	p	Multivariable analysis	p
	Estimates (95% CI)		Estimates (95% CI)	
ECOG	1.353 (1.003–1.824)	0.048*	1.133 (0.738–1.740)	0.567
Cancer Stage	0.550 (0.361–0.839)	0.006**	0.508 (0.293–0.880)	0.016*
Emotional distress (HADS)	1.187 (1.143–1.233)	<0.001***	1.090 (1.030–1.153)	0.003**
Insomnia severity (ISI)	1.093 (1.058–1.130)	<0.001***	0.907 (0.921–1.021)	0.246
Depression from PHQ-9	1.361 (1.279–1.448)	<0.001***	1.355 (1.227–1.497)	<0.001***
Health status (EQ-5D-5 L value)	0.096 (0.041–0.228)	<0.001***	1.696 (0.378-7.600)	0.490
Health status (EQ-5D-5 L VAS)	0.972 (0.962–0.982)	<0.001***	1.005 (0.989–1.021)	0.556
MDASI-pain	1.230 (1.136–1.333)	<0.001***	1.116 (0.980–1.272)	0.099
MDASI-fatigue	1.236 (1.142–1.337)	<0.001***	0.899 (0.778–1.038)	0.147
MDASI-upset	1.249 (1.151–1.356)	<0.001***	0.874 (0.760–1.004)	0.058
MDASI-forgetfulness	1.224 (1.125–1.332)	<0.001***	1.159 (1.028–1.306)	0.016*

***Abbreviations***: *ECOG- Eastern Cooperative Oncology Group performance status scale*.

Variables entered in the models: cancer stages, ECOG, HADS, PHQ-9, ISI, EQ-5D-5 L value and VAS, MDASI-pain, MDASI-fatigue, MDASI-upset, MDASI-forgetfulness.

***p<0.001; **p<0.01, *p<*0.05*.

### Mediation model of suicidal ideation in Chinese patients with advanced breast cancer

**Fig. 2:**
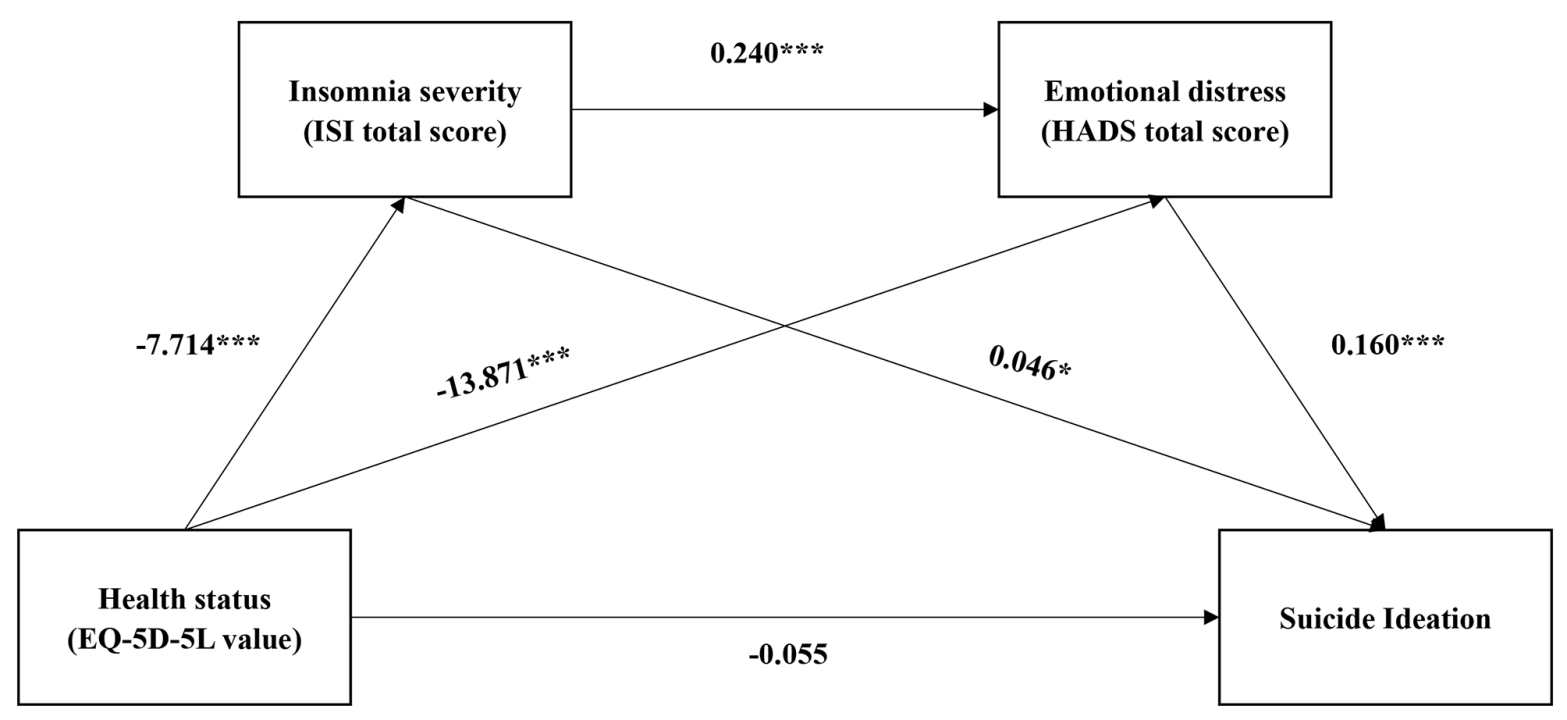
Serial mediating model and parameters of suicidal ideation in patients with advanced breast cancer

We used serial multiple mediation analyses to determine direct and indirect processes for predicting SI (denoted as Y) from physical and psychological variables in the study sample. MDASI-pain, MDASI-fatigue, MDASI-upset, MDASI-forgetfulness, emotional distress, depression, insomnia severity, and health status (assessed using EQ-5D-5L and VAS) were implemented separately as the independent variable (X) and the mediating factors (M) in the models. An acceptable model of SI was derived using the bootstrapping method, and is outlined as follows: health status (EQ-5D-5L value) was the independent variable (direct estimate = − 0.055, *p* = 0.547), insomnia severity (M1:indirect estimate = − 0.053, *p* = 0.054, adjusted R^2^ = 0.082) and emotional distress (M2:indirect estimate = − 0.333, *p* < 0.001, adjusted R^2^ = 0.284) were the mediating factors (M1 + M2:indirect estimate = − 0.044, *p* = 0.005). The total estimate effect was − 0.485 (*p* < 0.001). All parameters are presented in Table 4, and the mediating estimate effect among all the variables are outlined in Fig. 2.

**Table 4:** Parameters in the mediating model of suicide ideation (SI) in patients with advanced breast cancer

	Estimate	S.E.	z	p	Boot 95% CI		Effect size	
							R^2^	Adjusted R^2^
Indirect All	-0.430	0.056	-7.698	<0.001***	-0.542	-0.325	——	——
Indirect-X-M1-Y	-0.053	0.027	-1.928	0.054	-0.111	-0.005	0.084	0.082
Indirect-X-M2-Y	-0.333	0.047	-7.009	<0.001***	-0.431	-0.243	0.287	0.284
Indirect-X-M1-M2-Y	-0.044	0.016	-2.814	0.005**	-0.081	-0.021	——	——
Direct	-0.055	0.091	-0.602	0.547	-0.229	0.118	——	——
Total	-0.485	0.088	-5.481	<0.001***	-0.656	-0.326	——	——

***p<0.001; **p<0.01, *p<0.05

R^2^: small ≥ 0.02, medium ≥ 0.13, large ≥ 0.26

X = EQ-5D-5 L value; Y = Suicide ideation (SI); M1 = Insomnia severity (ISI total score); M2 = Emotional distress (HADS total score)

## Discussion

### Main results

Identifying suicide risk and addressing the mediating pathway from health status to SI among cancer patients is a crucial step in preventing suicide attempts and suicide. We found that depression, emotional distress, feeling upset, and forgetfulness are significant risk factors for SI in patients with advanced breast cancer. It was demonstrated that a low health status– mediated via insomnia and psychological distress– is significantly associated with SI in the study population. Known risk factors for suicide ideation include depression, anxiety, pain, and functional impairment [36, 37]. In particular, it has been established that depression is a key predictor of SI. Patients diagnosed with depression and other psychiatric disorders are at an elevated likelihood of being assessed for suicidal risk, although the findings of some research studies indicate that SI also occurs in individuals who do not meet the diagnostic criteria for depression. Alexopoulos et al. report that 7% of older community-dwelling adults have SI without depression and highlighted the importance of assessing this population [38]. Beyond their mental health history, the physical health and social support systems of cancer patients should be evaluated regularly. Joiner et al. propose that low family support and feeling like a burden can more effectively predict SI than depression alone [39]. Among breast cancer patients, additional risk factors include young age, male sex, single marital status, and surgery [40]. We emphasise a comprehensive suicide risk assessment for advanced breast cancer patients. Our findings reveal that there are between-group differences in the physical, psychological, and symptom burden variables for advanced breast cancer patients with SI versus without SI. Those with SI have worse physical function, are at a more advanced cancer stage, and experience higher distress across various measures. Furthermore, distant metastasis, emotional distress, depression, and cognitive impairment are independently predictive of SI. Consistent with our previous research, 27% of the advanced cancer patients who participated in this study reported a high multidimensional symptom burden [41]. Lage et al. propose that 40.2% of advanced cancer patients have functional impairment accompanied by severe physical and psychological symptoms [42]. Symptoms are a critical source of cancer distress and become a major risk factor for SI among patients with advanced cancer, which deserves considerable attention in clinical practice. However, most of the current research exploring the risk factors for SI are cross-sectional studies, which are inferior to longitudinal studies– especially prospective cohort studies. Franklin et al. identify five categories of risk factors for SI in their meta-analysis study: internal psychopathology, demographic factors, prior suicidal thoughts and behaviours, external psychopathology, and social factors [43]. For advanced cancer patients, we strongly recommend that their health condition be assessed thoroughly during systematic assessment for SI.

The variables considered in this study cannot exclusively affect the outcome of SI, as interactions among the variables and both direct and indirect effecting mechanisms are involved. The main contribution of this research is the derivation of a predicting model that captures all the effecting pathways. In the final model, insomnia severity and emotional distress mediate the relationship between health status and suicidal ideation. Previous studies have focused on the mediation effect of some psychological variables. For example, Zhou et al. report that family cohesion has both a direct and indirect effect on suicidal ideation among cancer patients [44]; psychological strain– mediated by hopelessness and psychological distress– is reported to influence suicidal ideation among stomach cancer patients [45]. Patients with advanced cancer experience a high symptom burden and a low health status. Hence, symptom management and improving quality of life are considered the main clinical tasks of palliative care providers who attend patients with advanced cancer. Therefore, investigating the mediating model between health status and suicidal ideation in this population provides further useful pointers for high-quality palliative care.

A breast cancer diagnosis and its treatment can negatively impact patients’ mental health, with a heightened risk for suicide as the most severe outcome, along with other outcomes such as anxiety, depression, sleep disturbance, stress-related disorder, and fear of cancer recurrence [46]. Although assisted suicide (AS) is legally permitted in certain contexts and personal autonomy around end-of-life decisions is increasingly supported [47], suicide remains an impulsive and emotionally traumatic event for patients, their families, and the medical staff who witness it. Proactive identification of at-risk patients and provision of rational psychosocial support are urgent priorities in clinical cancer care– especially end-of-life care. Furthermore, the waxing and waning trajectory of suicidal ideation provides a window for the early identification of suicidal tendencies and intervention to prevent suicide attempts [48, 49].

Although it was previously accepted that discussing suicide with patients would reduce, rather than increase, the patient’s suicidal ideation [50], it is still an emotionally difficult task to initiate dialogue about this issue. It has been recommended that Item 9 of the PHQ-9 be used independently to briefly assess suicidal ideation [51]. We used an ePRO platform that included this pertinent item in this multicentre study to avoid embarrassment for research associates with little psychiatric work experience, for timely identification of patients with suicidal ideation, and to automatically notify the psychiatrists on our research team to conduct further interviews with such patients.

### Study limitations

We must acknowledge the limitations of this study. First, while the cross-sectional study design allows multicentre sampling, it precludes determining causal relationships over time. As noted by Franklin, longitudinal data are optimal for elucidating suicide risk trajectories [36]. Although symptom burden and health status are identified as being predictive of SI in this study, cohort studies are required to confirm their risk effects. Second, our suicide risk evaluation– though multi-domain– was not exhaustive, per established frameworks; factors such as impulsivity, non-suicidal self-injury, and trauma history warrant inclusion given their potential independent, confounding, or moderating effects. Furthermore, highly comprehensive modelling of the advanced breast cancer population would strengthen prevention recommendations. We used a single subscale (item 9 of the PHQ-9) to briefly screen recent SI in patients with advanced breast cancer. The results may over or underestimate the real severity of SI compared to specific suicide assessment tools. In addition, the diversity of the study sample was limited, introducing generalizability concerns. A broader demographic sampling would provide more representative clinical guidance. Notwithstanding these limitations, which future research can address, our research findings highlight previously under-recognised predictors of suicidal ideation and provide useful information for improving integrated supportive care for Chinese patients with advanced breast cancer.

### Clinical implications

Screening and assessment of suicidal ideation are pivotal first steps in suicide prevention efforts in cancer care. The risk factors identified in this study should be directly considered in management protocols and interventions for suicidal ideation. The contents of comprehensive suicide assessment vary among different populations, and a tailored approach is warranted. Regarding advanced breast cancer, our research findings emphasise incorporating both symptom burden and psychological distress assessments with standard suicide risk evaluations. Comprehensive monitoring of the health status and quality of life of patients with advanced breast cancer could enable more responsive, supportive care for this population already facing profound physical, emotional and existential challenges. The data generated in this study can shape the development of highly effective suicidal ideation screening and treatment pathways with improved integration of oncology, palliative services, psychiatry, clinical psychology, and psychosocial support programming around patient-centred suicide prevention goals.

### Conclusion

SI has a high prevalence among advanced breast cancer patients, underscoring the need for comprehensive and integrated suicide prevention efforts. Multidimensional assessment of patients in this population should encompass symptom burden, health status, and psychological distress to guide targeted, patient-centred supportive care.

## Data Availability

The data supporting all the findings in this study are available from the corresponding author with reasonable request.

## Abbreviations

SI: Suicide Ideation
ePRO: electronic Patient-Reported Outcomes
PHQ-9: 9 item Patient Health Questionnaire
HADS: Hospital Anxiety and Depression Scale
ISI: Insomnia Severity Index
EQ-5D-5L: Five-level EuroQol five-dimensional questionnaire
MDASI: MD Anderson Symptom Inventory
WHO: World Health Organization
UN: United Nation
SMR: Standardized Mortality Ratio
PTSD: Post-Traumatic Stress Disorder
MDD: Major depression disorder
CIs: Confidence intervals
ECOG: Eastern Cooperative Oncology Group performance status scale
AS: Assisted suicide
SEER: Surveillance, Epidemiology, and End Results Program
